# Supplementary material for: Are carbon emissions trading and green financial instruments synergistic? -Comprehensive quantitative research based on content analysis
Source: PLoS One. 2024 Mar 7;19(3):e0298601. doi: 10.1371/journal.pone.0298601 (PMC10919601; doi:10.1371/journal.pone.0298601)
Supplement: S1 Data — (ZIP) [file pone.0298601.s001.zip › Data/地级市Z_F工作报告.zip!/╔╜╢1⁄2/║╩╘≤╩╨/└┤╫╘╣1⁄2╓┌║┼í╛┬φ┐╦╩2╛▌═°í┐_2008.docx]

各位代表:

　　现在,我代表市人民政府向大会作政府工作报告,请予审议,并请市政协委员和其他列席人员提出意见。

　　一、五年来的工作回顾

　　过去的五年,在中共菏泽市委的坚强领导下,市政府团结带领全市人民,认真贯彻落实科学发展观,全面实施“突破菏泽”战略,同心同德,干事创业,圆满完成了市十六届人大一次会议确定的各项目标任务,经济社会发展进入改革开放以来最好最快的时期。

　　经济综合实力明显增强。预计2007年实现生产总值653亿元,是2002年的2.6倍,年均增长16.1%。地方财政收入42亿元,是2002年的3.1倍,年均增长25.3%。各项存款余额572亿元,是2002年的2.1倍,年均增长15.9%。投资水平不断提高,发展后劲逐步增强。生产总值、工业主营业务收入、固定资产投资等主要经济指标增幅均高于全省平均水平,地方财政收入在全省前移一个位次。

　　经济运行质量显著提高。工业化进程明显加快,预计2007年工业增加值265亿元,占GDP的比重达到40.5%,比2002年提高14个百分点。2007年规模以上工业企业1305家,是2002年的4.2倍,预计实现主营业务收入900亿元、利税60亿元,分别是2002年的7倍、6倍。彭庄矿井正式投产,东明石化、菏泽电厂、供电公司、圣奥化工、达驰电气、洪业化工、玉皇化工、尚舜化工、步长制药等一批骨干企业迅速膨胀。服务业发展不断加快,预计2007年服务业增加值158亿元,是2002年的2.2倍;社会消费品零售总额326亿元,是2002年的2.1倍。三次产业结构由2002年的44:31:25将调整为28:48:24,二三产业比重提高16个百分点。科技进步对经济增长的贡献率达到43%。节能减排、环境保护、食品安全和节约集约用地取得积极成效,万元GDP能耗、COD排放量、SO2排放量持续下降。

　　新农村建设开局良好。不断加大“三农”投入,大力调整农业结构,推进产业化经营,农业综合生产能力有了新的提高。2007年粮食总产491万吨,棉花29万吨,肉类59万吨,森林覆盖率32%。规模以上农产品加工企业、省级以上农业龙头企业分别达到480家、17家。党和国家的支农惠农政策得到较好落实,全部免除了农业税,累计向农民发放补贴资金11亿元。实施了新农村建设“四四四一”工程,累计建设户用沼气池2.5万个,改路1.2万公里,改厕59.6万座,3265个行政村完成改水任务,受益人口288万人,农村生产生活条件进一步改善。预计农民人均纯收入3900元以上,年均增长11%以上。

　　改革开放取得较大进展。国有企业改革进一步深化,财政部门预算、国库集中支付、“乡财县管”全面推开,乡镇政府举债管理进一步加强。社会公益事业改革迈出新步伐,环卫管理等单位初步实现了“拿钱养人”到“拿钱办事”的历史性转变。小型水利设施和林业产权改革不断完善。对外开放水平进一步提高,五年累计实际利用外资5.5亿美元,2007年完成进出口总额7.8亿美元,是2002年的4.6倍。与美国、韩国、巴西、乌拉圭的四个城市结成了国际友好城市。企业上市实现了零的突破,单县尚舜化工、东明恒昌化工相继在新加坡挂牌上市,直接融资7.5亿元。成功举办了每年一度的菏泽国际牡丹花会和中国林产品交易会。

　　城市和基础设施建设实现重大突破。城市化水平达到30.5%,比2002年提高9个百分点。完成了新一轮《菏泽市城市总体规划》,城区控制性详规覆盖率达到85%。五年来累计投入财政性资金28亿元,带动社会投资84亿元,建设了环城公园、赵王河公园、市区引黄供水、污水处理、城区道路及综合配套管网等一大批重点工程,营建了环城林带和87处街头绿地、广场,建成商品房5.4万套,面积644万平方米,建成了中国林展馆、银座商城,城市综合开发力度不断加大。城市功能显著强化,城市特色初步显现,人居环境明显改善。九县区工业园被批准为省级工业园,园区功能不断增强。菏关、济菏高速公路建成通车,德商高速开工建设。累计改造国省道836公里,成为国家公路运输主枢纽城市,洙水河航道一期工程开工建设。黄河安全建设取得重大进展。对东鱼河南北支、鄄郓河及谢寨、闫潭送水干线等骨干河道进行了治理,防汛抗旱能力进一步提高。通信设施建设、电网建设及配电网改造等工作取得较好成绩。

　　和谐社会建设扎实推进。就业和社会保障工作得到加强,五年累计新增城镇就业40万人,农村在外务工人员达到140万人。城镇居民人均可支配收入9520元,年均增长11.3%。城镇职工基本养老保险、医疗保险和农村养老保险参保人数分别达到44万人、42万人和33万人,城乡低保人口分别达到5.4万人和25万人。全部免除了城乡义务教育阶段学生学杂费;累计投入危改资金7亿元,消除农村中小学危房146万平方米。实施了市区中小学校三年建设规划,已建成5所。组建了菏泽学院,成立了菏泽技术学院和菏泽家政职业学院,启动了菏泽职业技术学院筹备工作。疾病预防控制体系和传染病救治体系基本建成。新型农村合作医疗制度全面推开,参合率达到87.5%。文化事业健康发展,《山东汉子》荣获国家舞台艺术精品提名奖。安全生产和社会治安形势稳定。广播电视、新闻出版、计划生育、体育、人防、气象、地震、档案、史志、民族宗教、民兵预备役、老龄、妇女儿童和残疾人等各项事业都有了较快发展。

　　各位代表!过去的五年是我市经济快速健康发展,综合实力明显提升的五年;是社会事业全面进步,人民群众得到实惠明显增多的五年;是基础设施日益完善,城乡面貌发生明显变化的五年;是发展后劲明显增强,为实施跨越发展打下坚实基础的五年。五年来取得的成就,是省委、省政府实施“突破菏泽”战略的结果,是市委正确领导、市人大政协大力支持和社会各界有效监督的结果,是全市人民上下一心、奋发图强、扎实苦干的结果。在此,我代表市人民政府,向全市广大干部群众,向各民主党派、工商联、人民团体和社会各界人士,向驻菏部队、武警官兵和中央、省属单位,向所有关心支持菏泽发展的朋友们,表示诚挚的敬意和衷心的感谢!

　　在充分肯定成绩的同时,我们也清醒地看到,发展中还存在不少困难和问题,主要是:经济总量小,工业化水平低;自主创新能力不强,经济发展方式粗放;农业产业化进程缓慢,农民增收难度加大;节能减排任务艰巨,环境保护压力越来越大;土地紧张、资金短缺、人才匮乏仍是较长时期制约我市发展的重要因素;社会事业欠帐较多,社会保障能力不强,城乡部分低收入群众生活仍比较困难,不稳定因素大量存在;政府及其部门的工作在许多方面还存在差距,工作作风不够扎实,服务质量、行政效能都不够高,许多该给群众办的事情办得还不够好、不到位、不理想。对此,我们一定高度重视,采取有力措施认真加以解决。

　　二、今后五年的目标任务

　　未来五年是我市攻坚克难、奋力赶超、推动经济社会又好又快发展的关键时期。党的十七大为我们指明了前进方向;省委、省政府继续实施帮扶菏泽战略,加快鲁南经济带规划建设,为我市经济社会发展提供了重大战略机遇;经过近年来的艰苦努力,我市城市和基础设施条件显著改善,六大产业实力明显增强,一大批骨干企业快速崛起,特别是随着巨野煤田开发进程的加快和鲁南液体化工原料输送工程的实施,一批煤炭深加工和石油化工等大项目陆续开工建设,将极大地增强全市发展后劲和经济实力;全市上下人心思上,人心思干,人心思和。我们面临的机遇和有利条件比以往任何时候都好,我市经济社会发展已经站在新的历史起点上。我们一定坚定信心,抓住机遇,乘势而上,推动全市经济社会再上新台阶,再创新水平。

　　今后五年政府工作的总体要求是:认真贯彻党的十七大和省九次党代会精神,深入落实科学发展观,坚持稳中求进、好字优先、又好又快、科学发展,全力加快工业化进程,繁荣发展服务产业,努力提高城镇化水平,扎实推进新农村建设,着力构建和谐社会,努力实现全市经济社会科学发展、和谐发展、跨越发展。

　　今后五年全市经济社会发展的总体目标是:

　　———经济综合实力迈上新台阶。地区生产总值达到1300亿元以上,年均增长15%以上;规模以上工业企业主营业务收入3500亿元以上,比2007年基本翻两番;地方财政收入120亿元以上,年均增长24%以上,总量在全省的位次前移、跨入中等行列,人均与全省平均水平更加接近、与兄弟市的差距进一步缩小。

　　———产业结构进一步优化升级。三次产业比例调整到15:52:33,二三产业比重提高13个百分点。煤电化工基地、石油化工基地、农副产品生产加工基地、商贸物流基地初具规模。

　　———经济发展方式明显转变。自主创新能力不断增强,高新技术产业产值占比达到27%以上,科技进步对经济增长的贡献率达到50%以上。完成上级下达的节能减排、环境保护、安全生产目标任务。

　　———城乡面貌显著改善。城市功能更加完善,中国牡丹城和平原森林城市特色更加突出,城镇化水平达到40%,市区人口达到60万人以上,形成具有较强辐射带动能力的区域性中心城市。新农村建设取得重大进展。

　　———居民生活水平进一步提高。城乡就业更加充分,社会保障体系更加健全,家庭财产普遍增多,人民生活更加殷实。城镇居民人均可支配收入达到14600元,农民人均纯收入达到6000元,年均分别增长9%左右。

　　———各项社会事业全面发展。教育体系更加完善,全民受教育程度明显提高,公共文化服务体系建设取得较大进展,人人享有基本医疗卫生服务,社会管理体系更加健全。

　　五年看头年。今年是新一届政府履行职责的第一年,必须开好头、起好步。主要预期目标是:地区生产总值增长15%以上;规模以上工业企业主营业务收入增长30%以上,利税增长40%以上;固定资产投资增长22%;社会消费品零售总额增长16%以上;进出口总额增长16%以上;实际利用外资增长15%以上;地方财政收入增长25%以上;新增城镇就业11万人以上,登记失业率控制在3.8%以内;人口自然增长率控制在6.3‰以内;城镇居民人均可支配收入和农民人均纯收入均增长9%以上;居民消费价格总水平涨幅控制在4%左右;完成上级下达的节能减排、环境保护、安全生产年度目标。

　　各位代表!全面完成任期目标任务,责任重大,使命光荣。尽管前进道路上还会有很多困难和问题,但是我们坚信,有十七大精神的正确指引,有市委的坚强领导,有市人大、市政协的监督支持,有全市人民的团结奋斗,我们完全有能力战胜一切困难,圆满完成今后五年的奋斗目标,为全面建设小康社会打下更加坚实的基础!

　　三、转变发展方式,推动科学发展

　　把推进节约集约用地、提高节能环保水平、增强科技创新能力作为转变发展方式、推动科学发展的关键措施来抓。

　　(一)加强耕地保护和土地节约集约利用

　　严格耕地保护。进一步完善耕地保护目标责任制,认真执行问责制。继续巩固土地执法专项行动和百日行动成果。对新增用地严格按照法定程序报批,严肃查处土地违法违规行为。

　　加强新增用地管理。坚持项目联合审核制度,严把项目用地审批关,对新上投资项目,严格落实投资强度、建筑密度、建设容积率等控制性指标,确保把有限的土地指标用在最关键最需要的项目上。坚持有保有压、优中选优的原则,努力使一批大项目、好项目列为国家和省重点建设项目。建立健全工业用地公开出让机制,新增工业用地一律实行招拍挂。盘活存量土地。加强项目用地供后监管,对征而未用的依法予以收回,多征少用的进行整合,达不到投资强度的进行“瘦身”,或者限期追加投资。大力改造“空心村”,整治复垦废弃土地,清理整顿农村宅基地。积极抓好建设用地置换,搞好城镇建设用地增加与农村建设用地减少挂钩试点,拓宽建设用地空间。

　　(二)着力搞好环境保护

　　强化污染治理。今年确保84家省市控重点企业全部安装在线监测设备,并与县区以上环保部门联网。继续抓好治污配套设施建设,年内城市建成区管网覆盖率达到90%以上,污水收集率达到80%以上,污水处理厂满负荷运转率达到75%以上。三条主要河流出境断面水质稳定达到国家规定的水质标准。对20吨以上的燃煤锅炉强制安装脱硫设施和自动在线监测设备,完成二氧化硫排放削减任务,确保城区空气质量良好率达到95%以上。

　　严把新上项目环评关。加强环境总量控制,认真落实环境影响评价和“三同时”制度,防止新增污染。

　　严厉打击环境违法行为。加大监督检查力度,对不能稳定达标排放的企业或项目,予以限产、关停;对无故停运、不能稳定达标排放的污水处理厂,进行严肃查处;对河流出境断面水质连续三个月超标且无明显好转的县区,实行“区域限批”。

　　(三)大力推进节能降耗

　　提高重点企业节能水平。继续深入开展420户重点耗能企业对标节能活动,不断强化节能降耗措施,力争年内主要能耗指标达到行业标准或行业平均水平,其中300户企业达到省部级标准或同行业先进水平。

　　实施节能改造。加大节能降耗技术和产品研发力度,今年组织实施10项重点节能工程,100个节能降耗技术改造项目,30个节能产品研发。加强资源节约和综合利用,规划建设东明循环经济示范县、2个示范园和30家示范企业。积极推进区域集中供热和生物质发电等节能环保项目建设。

　　强化节能监管。严格执行节能评估审核制度,全面清理在建项目,严肃查处违法行为。全面落实节能目标责任制,对完不成节能任务目标的县区,实行“区域限批”和“一票否决”。

　　(四)提高自主创新能力

　　加强产学研合作创新。坚持项目、平台、人才一体化推进,今年重点抓好200家企业的产学研联合,完成新产品、新技术开发150项。突出抓好200家企业技术中心建设,争取60户重点企业全部按照省级标准建立企业技术中心或工程技术研究中心。鼓励企业加大研发投入,重点企业研发投入不低于销售收入的3%,高新技术企业达到5%。继续抓好创新型企业试点,今年新增国家和省级试点企业2-3家。下大力引进、培养一批高层次人才,五年内引进、培养100名职业经理人、100名博士、1000名硕士。

　　大力发展高新技术产业。坚持科技引领与投资拉动相结合,不断提高各级财政的科技支出比重,加大高新技术项目引进力度,今年高新技术投资占工业投资的比重达到20%以上,高新技术产业产值占规模以上工业的比重达到19%以上。着力培育医药化工、机电制造等特色产业集群,争取菏泽高新技术产业区尽早通过省级认定。

　　四、突出工作重点,推动跨越发展

　　充分利用我市资源、产业和区位优势,大力发展六大产业和商贸物流业,积极培育骨干龙头企业,强化基础设施支撑,争取通过五年的努力,初步建设成为重要的煤电化工基地、石油化工基地、农副产品生产加工基地、商贸物流基地。

　　(一)加快推进工业化进程

　　集中力量抓好大项目建设。一是加快在建项目建设。积极推进巨野煤田开发,确保龙固矿、赵楼矿今年上半年正式投产。争取全年煤炭产量达到600万吨。加快东明石化15万吨离子膜烧碱、洪业化工14万吨己二酸和10万吨环己酮、玉皇化工15万吨二甲醚、铁雄新沙150万吨焦化、圣世化工60万吨MCC、艺达家纺二期、华鹏玻璃三期、黄河纺织24万纱锭、天瑞纺织20万纱锭、天元纸业20万吨再生新闻纸等一批项目的建设进度,力争早投产、早见效。二是年内开工建设一批新项目。对于已经列入国家计划的赵楼煤矸石综合利用电厂、华润电厂、牡丹区和东明生物发电等电源项目,确保上半年开工建设。对于前期工作比较充分的万福矿、玉皇化工20万吨苯乙烯、肥矿120万吨焦化、洪业化工10万吨醇酮和16万吨己内酰胺、瑞丰搪瓷20万吨苯乙烯、圣奥化工5万吨扩建、巨润200万吨优质浮法玻璃等项目,争取尽早开工建设。紧紧抓住东明石化与中石油合作的机遇,尽快开工鲁南液体化工原料输送工程及东明石化120万吨催化裂解、30万吨石油焦综合利用、15万吨PVC、200万吨加氢精制等项目。三是储备一批项目。围绕培育六大产业,建设四大基地,加大项目策划力度。加快徐寨、孙寺等矿井勘探步伐,做好张集矿、陈蛮庄矿、电厂四期、里能郓城天然焦发电厂、郭屯电厂、东明石化扩能改造、洪业化工尼龙66盐、玉皇化工低碳烯烃、鲁麟矿业二甲醚、郓城中信焦化、达驰碳酸二甲酯、开发区高硫油加工、丰源甲醇等项目的前期工作,加快报批进度,争取早日批复。力争千万元以上的项目每年完成投资达到300亿元以上。

　　大力培育60户重点企业。进一步完善促进60户重点企业发展的各项政策措施,不折不扣地把财政税收、项目用地、融资担保、人才引进、自主创新、基础设施建设等政策落到实处。全面加强企业家队伍建设,尽快培养一批境界高、素质好、富有开拓创新精神、敢于干大事创大业的企业经营者。积极鼓励企业开展与国内外大企业、大财团的合资合作,实现快速膨胀。加强信用担保体系建设,鼓励企业之间开展互保、联保,多渠道解决企业贷款难题。继续加强银企合作,引导企业树立诚信意识;鼓励金融部门积极争取上级支持,创新金融产品和服务方式,加大对企业的支持,争取贷款增幅高于全省2个百分点以上。今年60户重点企业完成投资100亿元,实现主营业务收入400亿元,增长30%。

　　加大招商引资力度。进一步创新招商方式,紧紧抓住东南沿海地区特别是江浙沪产业转移的机遇,做好产业招商、以商招商的文章。加快市开发区中富等大型工业房地产项目建设,积极吸引更多有实力的建筑商开发工业房地产和商业房地产。精心组织好牡丹花会、林交会等各类招商活动。进一步完善园区基础设施、服务设施,强化园区功能,千方百计吸引大企业、好企业到园区投资。积极做好协调指导和服务工作,大力推动洪业化工、华信制药、达驰电气、步长制药、华意化工、龙泰果蔬等企业上市融资,确保今年4家企业成功上市。

　　(二)加快服务业发展步伐

　　改造提升传统商贸业。完成城市商业网点规划,按照规划要求,搞好市区商业网点建设,发展完善中华路城市商业中心,改造东方红商业街,推动同类经营店面集群经营。加快康庄乐园服装市场三期、香格里拉商贸城、曹县庄寨木材大市场、鄄城中药材大市场等十大特色专业市场建设。大力实施“万村千乡市场工程”和“农产品进超市工程”,积极引导流通企业开拓农村市场。

　　大力发展现代物流业。重点建设煤炭化工、石油化工两大物流园区。积极扶持菏泽交通物流、中邮物流、农资连锁等重点物流配送企业加快发展。鼓励有较大规模的工业企业剥离物流功能,发展第三方物流。加快口岸和保税物流中心建设,年内完成土地征用和铁路专用线报批工作。围绕建设国家公路运输主枢纽城市,加快运输场站建设,今年改建运输场站5个,改造农村客运站14个;全市客、货运周转量分别增长13%、12%。加快发展文化旅游业。加大牡丹、水浒、戏曲、武术等文化资源开发力度,发展特色文化产业。积极培育“一城三乡一生态”旅游品牌,着力塑造“中国平原森林城市”、“中国牡丹城”主体旅游形象,力争接待游客及旅游收入有明显增长,确保2009年实现创建中国优秀旅游城市目标。

　　扩大外贸进出口。加强农产品出口基地建设,引导企业调整优化出口商品结构,提高高新技术产品出口比重,努力扩大出口规模。用足用好国家鼓励进口的有关政策,做好原油、大豆、木材等原料进口工作。加快外派劳务基地县建设,积极开拓欧美、澳洲等发达国家劳务市场。争取到2012年进出口总额达到16亿美元,其中出口14亿美元,年均分别增长16%、15%。

　　努力发展商务服务业。积极发展金融保险业,创造条件,吸引股份制银行和证券公司来我市设立分支机构,积极争取村镇银行等新型农村金融机构试点。加快发展电子商务,重视发展科技、信息、财务、法律、咨询、房地产等中介组织,为经济发展提供全方位服务。

　　(三)加快新农村建设步伐

　　继续把解决“三农”问题作为重中之重,大力发展现代农业,繁荣农村经济,改善农民生产生活条件,努力增加农民收入。

　　加快发展农业产业化。围绕棉纺、食品、木材加工等优势产业,大力培育龙头企业。加大对30家重点龙头企业的政策扶持,通过新上一批高质量的项目,膨胀企业规模,提升市场竞争力和辐射带动力,今年主营业务收入达到65亿元,增长30%。加大农业结构调整力度,着力培育粮棉、果蔬、林木、畜牧等优势特色产业,促进区域化布局、规模化经营,建设一批与龙头企业相配套的优质农产品生产基地。鼓励更多的农民加入专业合作组织,建立和完善农村社会化服务体系。

　　大力推进农业科技进步。健全良种引进繁育推广体系,强力推进农业良种化,今年小麦、玉米良种率分别达到90%和95%,主要林木、畜禽良种率分别达到60%和75%。认真搞好良种良法配套,努力提高复种指数,积极推广林下养殖、林下种植等先进种养模式。建立农业技术市场,健全县乡村农业科技培训网络,实施科技特派员示范工程,为农民提供实用的技术服务。加强农业标准化建设,完善农产品质量标准、检测和认证体系,搞好动植物疫病防控,逐步提高农产品质量,确保达到国家强制性标准。

　　加强农业基础设施建设。加大财政对农业的投入力度,引导社会资金投向新农村建设。积极推进开发区东区水库、巨野大野水库建设,做好单县浮岗水库、曹县界牌水库除险加固工作。大力开展农田水利基本建设,重点抓好洙赵新河上游清淤治理,苏泗庄、闫潭等四大灌区节水改造,六条河道续建配套及6万亩涝洼地治理,提高综合防汛抗旱能力。不断加强农村通讯、电力、互联网建设。继续实施“四四四一”工程,年内新增改水受益人口100万人,改水普及率达到50%以上;新改建农村公路800公里,新建沼气池1.5万个。认真落实各项强农惠农政策。及时把粮食、良种、农资、农机等各项补贴政策不折不扣地兑现到农户。按照中央“三个明显高于”的要求,全面落实增加农业投入、加强农业基础建设的各项政策。切实稳定农村土地承包关系,依法维护农民承包土地的各项权利。

　　(四)加快城镇建设步伐

　　完善城镇规划设计。进一步理顺城乡规划管理体制,加强对县城的规划指导,完成县域村镇体系规划和乡镇总体规划。今年市区控制性详规覆盖率达到100%,县城达到70%以上。把城市设计放到更加重要的位置,重点抓好片区、项目和单体设计,打造更多设计精品。

　　抓好重点工程建设。今年城市建设任务十分艰巨,必须尽早启动。加快大剧院、演武楼建设进度,确保年底完成主体工程。完成赵王河新世纪公园和市垃圾处理工程。搞好曹州西路等5条主次干道改造及配套工程建设,完成丹阳路等10条道路供排水工程。开工建设城市规划展览馆。完善城市水系,打通环城公园西南进水口、赵王河南进水口和下游出水口,提高市区防洪排涝能力;加快外护堤河、赵王河、洙水河等截污工程建设,使城市水体彻底变清。搞好花城建设,在中华路、人民路、黄河路和牡丹路等9条城市主要干道上增植花卉、补植乔木,实施广州路、华英路等10项绿化工程,对现有绿地进行补植完善,达到三季有花、四季常青,丰富花城内涵,展示中国牡丹城特色。建设曹州路、环城公园二期等城市亮化工程。加快城市综合开发和“城中村”改造,重点抓好一批规模大、带动能力强的片区开发项目,今年房地产开发面积达到160万平方米以上。选取适当时机,规划建设市图书馆、艺术馆、博物馆、科技馆,完善城市功能,增强城市聚集能力。

　　提升城市管理水平。严格规划管理,严厉打击违法建设行为。加强环卫、绿化、亮化、公园等公共设施管理,综合治理城市脏乱及破坏市政设施的行为。加快发展城市公共交通,提高运营管理水平。年内完成城市交通管控信息系统建设,维护良好交通秩序。

　　加快基础设施建设。大力推进德商高速、鄄城黄河公路大桥建设,确保菏泽至曹县段年内通车。建设东新高速、菏枣高速,形成“米”字型高速公路框架。年内完成327国道巨野至菏泽段一级公路续建工程和省际边界公路改造任务。做好菏泽至兰考、菏泽至徐州地方铁路前期工作,争取早日开工建设。加快洙水河航道工程建设进度。进一步完善电网结构,年内开工建设3座220千伏和6座110千伏输变电工程。

　　五、着力改善民生,推动和谐发展

　　从人民群众最关心、最直接、最现实的利益问题入手,加大民生工程建设投入,促进社会和谐发展。

　　努力扩大社会就业。认真落实就业扶持政策,加强就业服务管理,确保年内各县区人力资源市场全部建成投用,搭建规范化的就业服务平台。加强农村劳动力转移培训、失业人员再就业及创业培训,以培训促进就业,以创业带动就业。开展就业援助,帮助困难群体尽快就业。力争每年培训6万人以上,新增城镇就业10万人以上,劳务输出18万人以上。

　　加快完善社会保障体系。进一步加大社会保险扩面征缴力度,确保企业离退休人员养老金和失业人员失业金按时足额发放。到2012年城镇基本养老保险参保率达到90%,农民养老保险参保人数明显增加。做好城镇居民基本医疗保险前期工作,2012年全社会基本医疗保险覆盖率达到75%。完善新农合制度,规范操作程序,提高补助标准,年内农民参合率达到90%以上。健全城乡低保制度,今年城市和农村低保标准分别提高到每人每月180元和每人每年900元。努力发展农村敬老事业,年内五保人员集中供养率达到70%。积极实施医疗、教育、减灾、慈善等救助救济,健全社会救助体系,逐步提高社会综合救助能力。发展老龄、残疾人和社会福利事业,做好优抚安置工作。加快经济适用住房和廉租住房建设,着力解决城市低收入家庭住房困难。今年全市开工建设经济适用住房25万平方米、廉租住房5万平方米。

　　优先发展教育事业。认真落实国家关于义务教育阶段的各项政策,加大对贫困家庭学生的资助力度。加快农村中小学危房改造,年内维修改造25万平方米,2009年前全部消除D级危房。年内全面完成10所城区学校三年规划建设任务。加强师资队伍建设,实施教师均衡配置工程,全面实施素质教育,提高教学质量,确保高考本科录取人数明显增长。支持菏泽学院、菏泽医专加快发展,提高菏泽家政职业学院和菏泽技术学院办学水平,今年完成菏泽职业技术学院一期工程,省级职业实习培训基地达到7处,全市中等职业教育招生3.5万人。

　　大力发展卫生事业。加大公共卫生投入,继续实施卫生支农工程;加强社区卫生服务,健全社区卫生服务网络。切实做好疾病预防控制工作,认真落实免疫规划政策,建立健全疫情报告处理机制。深入开展医疗惠民服务,积极增设平价门诊、平价病房。加快市立医院病房楼建设。打击医药购销和医疗服务中的不法行为,改进医德医风,努力解决群众看病难、看病贵的问题。广泛开展爱国卫生运动,积极发展妇幼卫生事业。鼓励中医药事业加快发展。严格落实人口目标管理责任制,稳定低生育水平,提高出生人口素质。

　　加快发展文化事业。大力发展农村和社区公共文化事业,着力抓好乡镇综合文化站、文化信息资源共享、农村电影放映、农家书屋等公共文化服务工程,争取春节前20户以上的自然村全部实现广播电视村村通。尽快启动鲁西南民俗文化村、影视娱乐城和市区大型公共文化设施建设。办好菏泽戏剧院,筹备成立市艺术团,着力打造一批精品剧目。抓好出版物市场和网吧市场监管,加强文物和非物质文化遗产保护。做好史志、档案工作。广泛开展全民健身活动,年内完成208处全民健身工程;提高竞技体育水平,做好承办十一届全运会散手比赛的各项筹备工作。

　　扎实推进平安建设。强化社会治安综合治理,深入开展严打整治斗争,着重加强社区和农村警务,健全打防管控一体化长效机制。大力加强对敌斗争,严打“法轮功”等邪教组织。完善和改进信访工作,认真排查解决群众反映的热点难点问题。建立完善快速反应机制,及时妥善解决群体性上访事件。深入开展“五五”普法活动,增强全民法制意识。做好民族工作,增进民族团结。

　　切实抓好安全生产。坚持“一岗双责”,加强基层基础工作,严格落实政府监管责任和企业主体责任,深入开展消防、交通运输、建筑施工、烟花爆竹、危险化学品等专项整治。完善落实安全生产预案,提高事故预防和应急救援能力。力争杜绝重特大安全事故发生,维护好群众的生命财产安全。

　　全面提高食品药品安全水平。积极推进质量认证,定检产品质量合格率达到90%以上,食品药品全部达到国家强制性标准。健全食品药品监管和责任追究体系,严格市场准入,强化市场监管,建立长效机制,切实保障食品药品安全。

　　努力增加城乡居民收入。在发展农村经济、增加农民收入,加强社会保障、提高困难群众生活水平的同时,全面落实企业工资支付规定和工资指导线制度,建立企业职工工资正常增长机制和支付保障机制。随着财政状况的好转,不断提高机关事业单位工作人员的工资津贴标准,确保每年都有一定幅度的增长,逐步缩小与发达地区的收入差距。

　　六、扎实推进服务型政府建设

　　面对艰巨繁重的发展任务,我们一定按照为民、务实、清廉的要求,进一步加强政府自身建设,全面提高行政能力和服务水平。

　　(一)进一步解放思想。认真学习贯彻党的十七大精神,坚持用马克思主义中国化的最新成果武装头脑,指导实践,推动工作。联系我市实际,努力做到“四破四立”:破除不重视学习、凭经验办事、固步自封的观念,树立勤奋学习、善于思考、勇于创新的观念,增强新形势下指导工作、破解难题的能力和水平;破除小成即满、小富即安的观念,树立干大事、创大业的观念,始终保持永不满足、奋发有为的精神状态,争创一流的工作业绩;破除只重速度、忽视效益,只要增长、牺牲环境,只重当前、不顾长远的观念,树立稳中求进、好字优先、又好又快、永续发展的观念,着力解决影响和制约科学发展的突出问题;破除重监管、轻服务,只求单位利益、忽视全局利益的观念,树立全市一盘棋、整体利益至上的观念,自觉服从服务于全市发展大局,齐心协力营造良好的发展环境。

　　(二)切实转变工作作风。严格实行目标管理责任制,对全年经济社会发展目标逐项细化分解,责任到人,形成一级抓一级、一级对一级负责的目标责任体系。进一步精简会议,压缩文件,减少应酬,提高效率,把更多的精力和时间用在解决热点难点问题上,用在深入基层调查研究上,用在推动工作措施落实上。特别是在一些重点领域、关键环节和重大问题上,各级政府及其部门主要负责人要负总责、亲自抓、带头干,加强检查,跟踪调度,直至问题解决。健全考核体系,完善考核办法,严格兑现奖惩,严肃查处弄虚作假、虚报浮夸行为。

　　(三)积极推进依法行政。自觉接受人大、政协和社会各界的监督,认真办理人大代表建议和政协委员提案,高度重视民主党派、工商联、无党派人士和人民团体的意见。加强基层民主建设,保证人民群众依法行使民主权利。健全重大决策事项社会公示制度、社会听证制度和专家咨询制度,进一步提高政府科学决策、民主决策水平。严格按照法定权限和程序行使权力、履行职责,依法管理经济社会事务。强化行政执法责任制,严格实行问责制。认真做好行政复议工作,强化审计监督、行政监察,严肃查处各类违法乱纪行为。

　　(四)着力优化服务环境。加快推进政企分开、政事分开、政资分开、政府与市场中介组织分开,切实把政府经济管理职能转到主要为市场主体服务和创造良好发展环境上来。继续深化行政审批制度改革,减少审批事项,简化审批程序,规范审批行为,健全行政审批中心运行机制,提供高效便捷服务。深入推进政务公开,加强电子政务建设,保障广大群众的知情权、参与权、监督权。充分发挥市长公开电话、纠风办、行政效能监察投诉中心等职能作用,进一步加大“三乱”治理力度,切实解决损害群众利益、破坏发展环境的突出问题。

　　(五)大力加强廉政建设。认真落实党风廉政建设责任制和领导干部廉洁自律若干规定,自觉规范领导干部的从政行为。深入开展治理商业贿赂工作,在工程招投标、土地出让、房屋拆迁、项目建设、产权交易、政府采购等关键领域,加大从源头上治理腐败工作力度,着力构建有效的监督机制。深入开展反腐败斗争,依法严肃查处违法违纪案件,坚决惩处各类腐败分子。牢记“两个务必”,大力倡导勤俭节约、勤俭办一切事业,坚决反对铺张浪费,规范公务接待,树立清正廉洁的良好形象。

　　各位代表!欠发达不是菏泽的专利。未来五年全市人民奋发图强,必将使菏泽大地发生沧桑巨变。让我们高举旗帜,团结奋斗,科学发展,大步跨越,早日改变菏泽欠发达面貌,让人民群众过上更加美好的生活!
